# Simulation and Experimental Research on the Charged Characteristics of Particulate Matter in the Sensor under Different Exhaust States

**DOI:** 10.3390/s20216226

**Published:** 2020-10-31

**Authors:** Dong Tang, Zhixuan Ju, Li Wang

**Affiliations:** Department of Automotive and Traffic Engineering, Jiangsu University, Zhenjiang 212013, China; 2221804008@stmail.ujs.edu.cn (Z.J.); 2221704016@stmail.ujs.edu.cn (L.W.)

**Keywords:** particulate, particulate sensor, charged characteristics, numerical simulation

## Abstract

The particulate matter sensor needs to be used in order to detect the concentration of particulate matter in diesel engine exhaust, monitor the working condition of diesel particulate filter (DPF) in real time, and ensure the reliable operation of DPF. The flow field and electric field of the sensor are studied and their distribution in the sensor is analyzed. At the same time, the particle tracking model was used to simulate the charged characteristics of particles in the sensor under different exhaust states. It is found that the exhaust gas flow rate maintains stability after entering the outer protection zone and concentration test zone. The electric field is a non-uniform electric field and the direction of electric field intensity is from the high voltage electrode to the grounding electrode. The electric charge per particle will decrease with the increase of exhaust flow rate, but the electric charge shows a slow growth trend. The charge of particles increases with the increase of exhaust temperature, exhaust gas concentration and particle size. The study of the charged characteristics under different environmental conditions provides a theoretical basis for further improving the prototype mechanism of a leakage flow particle sensor.

## 1. Introduction

**Figure 1:**
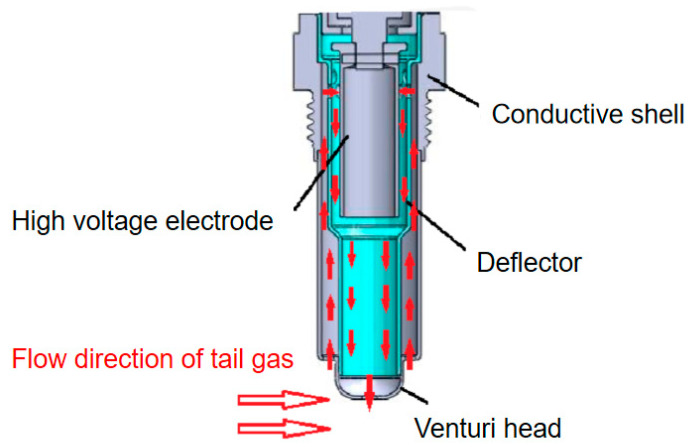
Structure of leakage current particulate matter sensor.

The diesel engine has been widely used in construction machinery, agricultural machinery, and transportation field because of its excellent power and fuel economy. The particulate matter (PM) emission cause by diesel engines has a great impact on the human health and environment [1,2,3], and it has an important impact on the generation of haze phenomenon. At present, the main way to remove the particles is to install diesel particulate filter (DPF) on the diesel engine [4,5], whose efficiency can reach more than 90%. However, the on-line diagnosis system (OBD) is needed for the reliable monitoring of DPF. With the increasingly strict requirements of OBD emission regulations, the error of traditional differential pressure sensor may exceed the limit value itself [6]. Therefore, a new type of sensor that can measure the low concentration particulate matter in real time is urgently needed [7,8,9]. At present, the widely used particle sensor is a resistance type particle sensor jointly developed by Bosch Company of Germany and NTK company of Japan. It adopts multilayer ceramic technology to convert the resistance value into the concentration of particles [10,11,12]. However, the real-time concentration of particles cannot be obtained because the resistance value is an accumulation value. Therefore, Almendinger et al. proposed a particle sensor [13] for measuring particle concentration and charge loss, as shown in Figure 1. As the exhaust gas flows through the sensor, there is relatively low air pressure above the leeward side of the sensor inlet, which causes the exhaust gas to flow into the sensor. There is a high voltage electrode on one side of the concentration test range. The particles will be ionized or polarized under the action of high voltage direct current, and move towards the ground electrode under the action of electric field for charge transmission, thus forming a leakage current between the electrode and the conductive shell. The value of the leakage current changes with the change of particle mass concentration in the exhaust gas. The concentration of exhaust particles can be obtained by collecting the leakage current generated by particle movement between electrodes [14,15].

Based on the principle of corona discharge, Russell established the principle of electric field charge in 1923, proving that particles are charged by collision with ionized ions. With the deepening of people’s understanding of the charging process of electric neutral objects, the theoretical research results of particle charging have been widely applied to production and life, electrostatic precipitator is the typical application direction of electrostatic field research on the effect of particles.

Domestic and foreign researches have been carried out on the complex environment of internal flow field, temperature field, particle field, electric field, and other fields under the action of multi-phase coupling in the high-temperature dust removal process of a dust collector. Luo et al. found in their study that temperature change would enhance the effect of ion wind in the sensor and increase the turbulence of airflow inside the dust collector. At the same time, the viscosity of the gas would also increase with the increase of temperature, inhibiting the movement of particles and affecting the dust removal efficiency [16]. Wu Xiaojun simulated and analyzed the electric field, flow field and particle distribution inside the dust collector, studied the charging, movement and deposition rules of particles under the coupling effect of multiple physical fields, and analyzed the influence rules of working voltage, exhaust velocity, particle size, and sensor structural parameters on the dust removal efficiency of the dust collector [17].

The output signal value of the leakage current particulate matter sensor is mainly based on the charge transmission of particles in the sensor concentration test range. The particle concentration is the main factor affecting the output signal value of the sensor. However, in the actual working environment, parameters such as exhaust flow rate, exhaust temperature and particle shape will have a positive impact on the charge characteristics of particles and the charge transfer between the sensor plates, thus affecting the sensitivity and accuracy of the output signal value of the sensor.

In this paper, through the numerical simulation of the charged characteristics under different environmental conditions, the influence of exhaust flow rate, exhaust temperature, and particle size on the charge of particles in the sensor is analyzed, which provides a theoretical basis for further improving the prototype mechanism of the leakage flow particle sensor.

## 2. Establishment of Numerical Model

### 2.1. Physical Field Model

#### 2.1.1. Gas Phase Flow Model

The particles flow into the particle sensor through the exhaust pipe, and the flow state is mainly affected by the fluid action. The fluid is regarded as a stable and incompressible state, and the fluid movement can be regarded as irregular turbulent movement [18]. The turbulent motion mainly satisfies the following conservation equations of mass and kinetic energy:

Conservation of mass equation:

(1) $$\frac{\partial p}{\partial t}+\frac{\partial }{\partial \chi_{i}}(\rho u_{i}^{})=0$$

Conservation equation of momentum:

(2) $$\frac{\partial p}{\partial t}(pu_{i})+\frac{\partial }{\partial x}(pu_{i}u_{j})=-\frac{\partial p}{\partial x_{i}}+\frac{\partial }{\partial x_{j}}(\mu \frac{\partial u_{i}^{}}{\partial x_{j}}-pu_{i}^{\prime }u_{{}_{j}}^{\prime })$$

where ρ is the fluid density, *u_i_* = (*u*_1_, *u*_2_, *u*_3_) is the average velocity component, *xi* = (*x*_1_, *x*_2_, *x*_3_) is the Cartesian coordinate, and *p* is the average pressure.

Common turbulence models include Spalart–Allmaras model, large eddy simulation model (LES), and Reynolds stress model (RSM). In this paper, the turbulence model is selected to analyze the flow field in the sensor to obtain the fluid distribution characteristics in the particle sensor [19]. The calculation equation is as follows:

(3) $$\frac{\partial (\rho k)}{\partial t}+\frac{\partial (\rho k\mu_{t})}{\partial x_{j}}=\frac{\partial }{\partial x_{j}}[(\mu +\frac{\mu_{t}}{\sigma_{k}})\frac{\partial_{\varepsilon }}{\partial x_{j}}]+G_{k}-\rho \varepsilon$$

(4) $$\frac{\partial (\rho k)}{\partial t}+\frac{\partial (\rho \varepsilon \mu_{t})}{\partial x_{j}}=\frac{\partial }{\partial x_{j}}[(\mu +\frac{\mu_{t}}{\sigma_{\varepsilon }})\frac{\partial \varepsilon }{\partial x_{j}}]+\frac{C_{1}\varepsilon^{\varepsilon }}{k}G_{k}-C_{2\varepsilon }\rho \frac{\varepsilon^{2}}{k}$$

GK in Equation (4) is the production term of turbulent kinetic energy *k* caused by average velocity gradient, the calculation formula is as follows:

(5) $$Gk=\mu_{t}(\frac{\partial u_{i}}{\partial x_{j}}+\frac{\partial u_{j}}{\partial x_{i}})\frac{\partial u_{i}}{\partial x_{j}}$$

In Equations (3)–(5), *k* is turbulent kinetic energy, $\mu_{r}(Pa\cdot s)$ is turbulent viscosity coefficient, and $\sigma_{k}$, $\sigma_{\varepsilon }$ is the turbulent Prandtl number of *k* equation and ε equation.

#### 2.1.2. Particle Tracking Model

Particles exist in a discrete form in the particulate matter sensor. The motion of particles is described by Lagrangian method. Particles are affected by multiple forces due to the joint action of flow field and electric field. Considering that the volume fraction of particles in exhaust gas is about 5%, and the distance between particles is greater than twice the diameter of particles, the collision of particles is not considered. In diesel engine exhaust, buoyancy, pressure gradient force, Magnus force, Brazil special force, and apparent mass force can be ignored since the density of particles (the density of carbon particles is about 3000 kg/m^3^) is far greater than that of gas [20].

In the COMSOL multiphysics software, the fluid particle tracking module is selected. This module includes two phases: discrete phase composed of particles and continuous phase of immersed particles which is air in this paper. The motion of particles follows the Newtonian equation of motion, and the kinetic energy equation of particle phase is as follows:

(6) $$\frac{d(m_{p}v)}{d_{t}}=F_{t}$$

At the same time, it is affected by traction, gravity and electric field force exerted by high-voltage electric field. The gravity of particles is:

(7) $$F_{G}=m_{p}g\frac{\left(\rho_{p}-\rho \right)}{\rho_{p}}$$

where: *m_p_* is the mass of particles; *g* is the acceleration of gravity; ρ is the density of exhaust gas; $\rho_{p}$ is the density of particles.

The drag force of particles is:

(8) $$F\tau =\frac{1}{\tau_{p}}m_{p}(u-v)$$

(9) $$\tau_{p}=\frac{p_{\rho }d_{p}^{2}}{18\mu }$$

where $\tau_{p}$ is drag coefficient, *u* = *u* (*x*, *y*, *z*, *t*) is fluid velocity, *v* = *v* (*x*, *y*, *z*, *t*) is particle velocity, *d_p_* is particle diameter, and μ is dynamic viscosity.

The electric field force of particles is:

FE = eZE(10)

where e is the charge quantity of element charge; Z is the number of charge carried by particles; E is the electric field strength.

#### 2.1.3. Steady State Electrostatic Field Model

The high voltage electrode in the concentration test area of the particulate matter sensor is connected to the power supply and the grounding electrode is grounded through the sensor shell leading to a steady-state electric field formed in the concentration test area. The steady-state electrostatic field model is adopted in the COMSOL multiphysics software, the electric field strength is 1000 V, and the electric field strength distribution is:

(11) $$\vec{E}=-\nabla \phi$$

where $\vec{E}$ is the electric field strength and ϕ is the discharge potential. As the amount of charge of particles entering the sensor is small, the electric field generated by the particles has little impact on the original high-voltage electric field.

### 2.2. Particle Charging Mechanism

There are usually a small number of ionized gas molecules and free electrons in natural space. When a high enough voltage is applied to the sensor electrode, an uneven electric field is generated in the discharge space when the discharge electrode reaches a certain halo voltage. The electric field intensity in the corona region near the electrode is much higher than that in other parts. Under the action of electric field, free electrons in the corona region will accelerate their movement and collide with gas molecules to further generate electrons and charged ions. Newly generated electrons will continue to collide with gas molecules. In the continuous corona discharge process, new electrons and charged ions will be continuously generated, which is the phenomenon of corona discharge. After escaping from the corona area, charged ions move towards the low potential area under the action of electric field force. When the particle size is larger than 0.5 μm, the charge of the particle is mainly electric field charge. Charged ions will collide with the particle and then be continuously adsorbed on the surface, making the particle have the same electrical properties as charged ions. When the charge of particulate increases to a certain degree, charged ions will no longer continue to be absorbed, reaching the saturation charge. When the particle size is less than 0.2 μm, the diffusion charge is the main charge for the particle, and the charged ion adsorbs the charge by collision with the particle through irregular thermal motion.

Based on the working principle of the leakage current particle sensor, the high-voltage electrode in the concentration testing area of the sensor will generate corona discharge, which will continuously charge the fine particles flowing into it, thus affecting the leakage current value and the accuracy of the output signal value of the sensor.

#### 2.2.1. Electric Field Charged

For the particles moving in the dc electric field, assuming that they are only affected by the electric field charge, the amount of charge obtained by the particles in time T can be calculated by the following formula:

(12) $$q_{f}=3\pi \varepsilon_{0}Ed_{p}^{2}\left(\frac{\varepsilon }{\varepsilon +2}\right)\left(\frac{1}{1+\frac{\tau_{q}}{t}}\right)$$

where $\tau_{q}$ is the time constant; *E* (V/m) is the electric field intensity in the charged area; *d_p_* (m) is particle diameter; $\varepsilon_{0}$ (8.85 × 10^−12^ F/m) is the vacuum dielectric constant; T (s) is time; ε is the relative dielectric constant of particles and it can be calculated by the following formula:

(13) $$\varepsilon =\frac{4\pi \mu d_{p}}{k_{b}T\rho_{r}\varepsilon_{0}}$$

where μ (kg/(s·m)) is the dynamic viscosity of the medium around the particle; *k_b_* (1.38066 × 10^−23^ J/K) is Boltzmann constant; T (k) is the absolute temperature of flue gas; $\rho_{r}$ (Ω m) denotes the particulate matter specific resistance.

The dynamic viscosity is related to the temperature of the gas and increases with the temperature of the gas. The influence of gas pressure on dynamic viscosity can be ignored under normal pressure. Under normal pressure, the relationship between the aerodynamic viscosity and temperature T can be expressed empirically as follows:

(14) $$\mu =\mu_{0}\frac{273+C}{T+C}{\left(\frac{T}{273}\right)}^{1.5}$$

where $\mu_{0}$ (kg/(s.m)) is the dynamic viscosity of gas at 0 °C and *C* is the constant related to gas properties. For air, the dynamic viscosity is 17.16 × 10^−6^ and the constant *C* is 122.

Generally, time constant q is far less than *t* (*t* refers to the duration of the particle’s stay in the charged electric field). It is usually assumed that the particle quickly reaches the saturation charge. The particle electric field charge can be simplified as follows:

(15) $$q_{f}=3\pi \varepsilon_{0}Ed_{p}^{2}\left(\frac{\varepsilon }{\varepsilon +2}\right)$$

#### 2.2.2. Diffusion of Charged

The particles move thermally and diffuse through the gas, colliding with the particles in the fluid and charging them. The charge depends mainly on the kinetic energy of the ion thermal motion, particle size and charge time.

The theoretical equation of diffusion charge is:

(16) $$q_{k}=\frac{\pi \varepsilon_{0}kTd_{p}}{e}\ln(1+\frac{e^{2}N_{0}d_{p}t}{2\varepsilon_{0}\sqrt{2m\pi kT}})$$

where *e* (1.6 × 10^−19^ c) is the electron charge, *N*_0_ is the spatial ion density, and *m* (5.3 × 10^−26^ kg) is the particle mass, where:

(17) $$N_{0}=\frac{j}{ekE}$$

where *j* (A/m^3^) is the measured emission current surface density, and K (m^2^/(s·V)) is the ionic mobility. Ion mobility mainly depends on the temperature and pressure of electric field, and its calculation formula is as follows:

(18) $$k=k_{0}\sqrt{\frac{T}{T_{0}}}(\frac{1+\frac{S}{T_{0}}}{1+\frac{S}{T}})\frac{P_{0}}{P}$$

where *k*_0_ (m^2^/(s·V)) is the ion migration rule of a gas in the standard state, T (K) is the absolute temperature in the actual situation, *T*_0_ (K) is the actual temperature in the standard state, *S* is the Surtherland constant of the gas, *P*_0_ (Pa) is the pressure in the standard state, and *P* (Pa) is the pressure in the actual situation.

The size distribution of diesel exhaust particles ranges from 0.01 μm to 10 μm, making particles simultaneously affected by the combined action of electric field charge and diffusion charge in the electric field. Using the method proposed by Robinson [21], it can be regarded as the joint action of two charging mechanisms, and the charge can be expressed approximately by the sum of the two:

(19) $$q=q_{f}+q_{k}$$

### 2.3. Geometric Model

**Figure 2:**
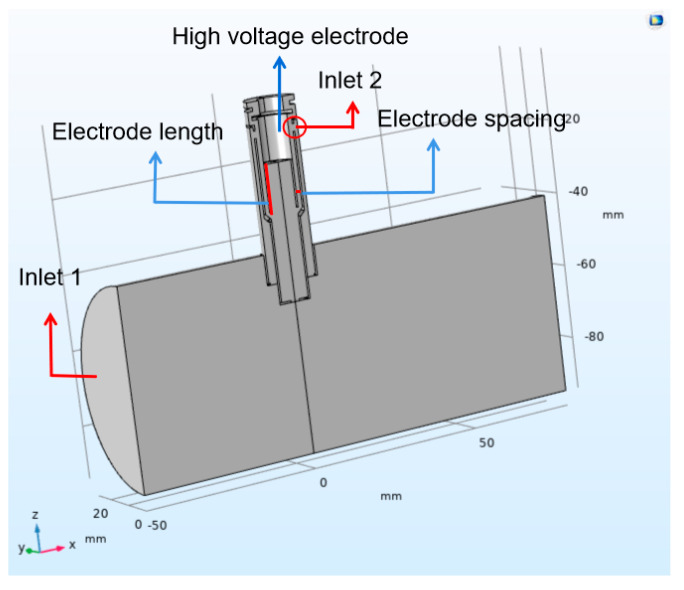
Model of leakage current particulate matter sensor.

The geometric model of the leakage current particulate matter sensor studied in this paper is shown in Figure 2. Due to the velocity and pressure of the gas inside the sensor are affected by the length of exhaust pipe and the installation position of the sensor, a three-dimensional model of the sensor installed on the exhaust pipe is established. As the sensor is an axisymmetric structure, only half of the model is established in order to reduce the calculation amount of numerical simulation. The charge of the particles is mainly carried out in the concentration test area, the distribution of the physical field in the sensor, the charged characteristics and the motion state of the particles are affected by the structural parameters of the concentration test area, thus affecting the output signal value of the sensor. The electrode length and the electrode spacing is set as 12.5 mm and 1.25 mm, respectively, while setting the inlet of exhaust pipe as inlet 1 and the inlet of concentration test section as inlet 2.

### 2.4. Boundary Conditions

#### 2.4.1. Gas Phase Boundary Conditions

The boundary conditions of the gas phase are set as follows. Air is selected as the fluid material. Velocity is adopted as the inlet boundary. 5 m/s, 10 m/s and 15 m/s are selected as the inlet velocity of intake pipe, according to the distribution of exhaust velocity at low load of four-cylinder diesel engine. Considering the actual exhaust temperature of the diesel engine at low load, 50, 100, and 150 °C are selected as the temperature of the fluid, respectively. Because of the complex velocity distribution near the wall, the wall function is selected as the fluid boundary condition. Turbulence intensity and turbulence length are chosen to adjust the turbulence condition, because the gas flow has been fully developed in the exhaust pipe.

#### 2.4.2. Particle Phase Boundary Conditions

For particles, escape conditions are used in the inlet and outlet. Velocity and pressure are respectively set as the conditions of inlet and outlet. Particles are uniformly released at the inlet. Rebound is selected as the wall condition. The size range of diesel engine particle is located in 0.01–1 μm [22,23], however, most of the particles are with smaller particle size. In order to simulate the charge characteristics of particles with different sizes, particles with the size of 20, 100, and 150 nm are studied for simulation analysis. Because most of the exhaust particles are charged with 1–5 units, the movement of particles with +3 units charge is studied in this paper.

## 3. Coupling Distribution of Multiple Physical Fields in the Sensor

### 3.1. Simulation of Flow Field Distribution in the Sensor

**Figure 3:**
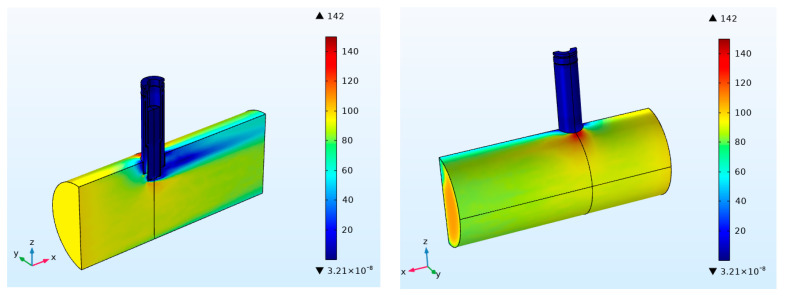
Speed distribution in sensor and exhaust pipe.

The movement state of particles depends on the distribution of flow field in the sensor. Mastering the distribution law of flow field in the particle sensor is conducive to further analyzing the measurement accuracy and sensitivity change of the sensor. The distribution of flow field with different flow rates is almost the same. Therefore, the flow rate at the inlet of exhaust pipe is set as 100 m/s, and the simulation results are shown in Figure 3.

As shown in the Figure 3, no obvious change occurs in the speed distribution when the exhaust gas does not flow through the sensor. The flow rate is basically maintained at 100 m/s. When the exhaust gas comes into contact with the sensor, the exhaust gas velocity decreases significantly. The exhaust flow velocity at the upper end of the leeward side drops to 20 m/s and then gradually increases. When the exhaust reaches the outlet of the exhaust pipe, the exhaust flow velocity slowly increases to 80 m/s. A little variation in the exhaust flow velocity occurs at the lower end of the sensor leeward side. Therefore, an obvious flow boundary layer appears. The exhaust flow velocity near the exhaust pipe wall slightly decreases. In all, the maximum value of the flow velocity can reach 142 m/s, which appears at the lower end of the sensor inlet, and the junction of the sensor side and the exhaust pipe. The flow velocity inside the sensor is relatively stable. The flow velocity in the concentration test area is below 20 m/s.

### 3.2. Simulation of Electric Field Distribution in the Sensor

**Figure 4:**
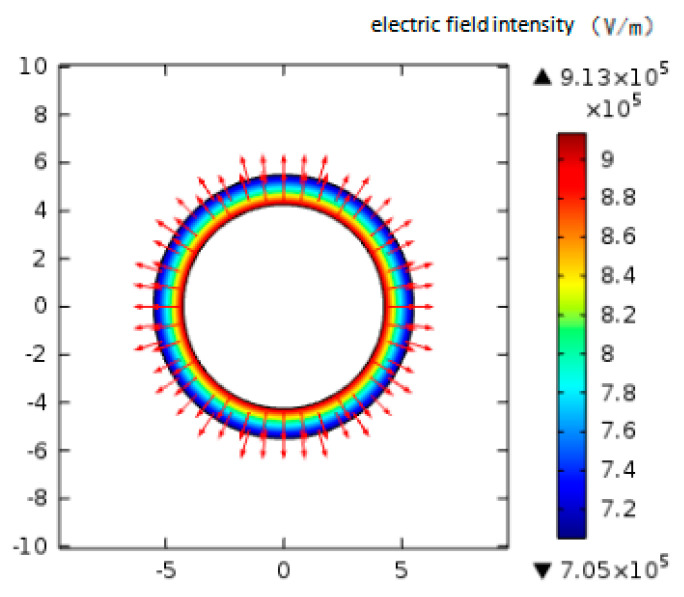
The distribution of electric field intensity on the section of particle concentration measurement.

A 1000 V high-voltage electrode and the shell connecting ground are respectively placed on two sides of the concentration test zone in the sensor, which forms a non-uniform strong electric field. The particles flowing in the interval are charged, which influences their motion state to complete the charge transfer and generate a leakage current signal. The electric field intensity distribution on the cross section of the concentration test zone is shown in Figure 4. The electrode length and the electrode spacing are 12.5 mm and 1.25 mm, respectively.

As shown in Figure 4, the electric field strength gradually decreases from the high-voltage electrode to the ground electrode. The charged particles are attached to the electrode with high field strength and fully charged. The amount of charge continues to increase with the accumulation and charging of particles. The electric field force moves the agglomerated particles against the adhesion to the ground electrode. At this time, the particles release the charge to generate a signal. In order to ensure the full charge of the particles and the sensitivity of the sensor output signal value, the electric field should maintain enough strength.

## 4. Analysis of the Influencing Factors of the Particle Charging Characteristics

Based on the above-mentioned particle sensor model, the simulation of the charged characteristics of exhaust particulate matter under the coupling effect of multi-physics is conducted. To be specific, environmental temperature, exhaust flow rate, and particle size are focused on to analyze the change rule of particle charge.

### 4.1. Exhaust Temperature

The normal inflow velocity at the inlet 1, the electrode voltage, the concentration of particulate matter and its size are set as 10 m/s, 1000 V, 1.6 mg/m^3^, and 100 nm. The law of the charge of the particulate matter flowing through the sensor at 50, 100, and 150 °C is studied.

**Figure 5:**
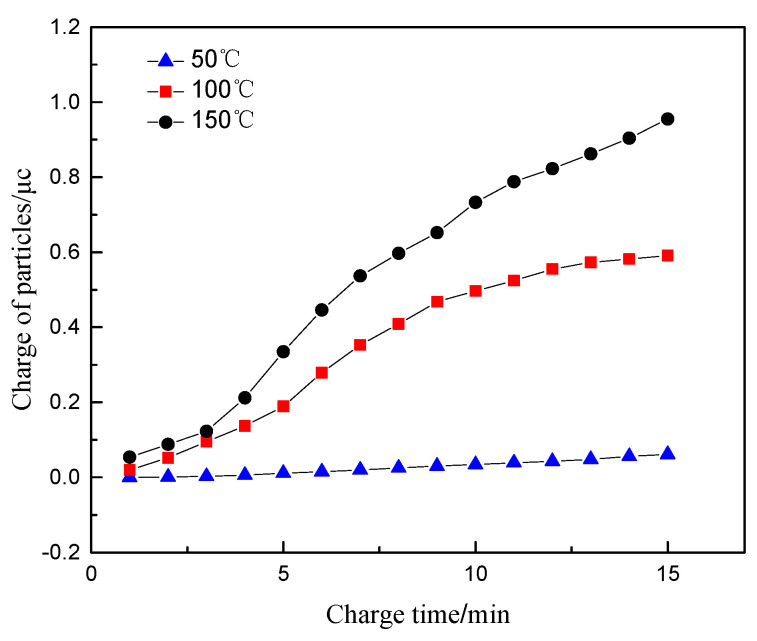
The relationship between the charge of particles and time at different temperatures.

Figure 5 shows the variation of the charge of particulate matter with time at different temperature. It can be seen that at the temperature of 50 °C, the charge of particulate matter does not change significantly with the increase of the charge time. This is because when the temperature is low, the high-voltage electrode does not reach the corona voltage, and thus the corona discharge is insufficient, which leads to the particulates failing to be effectively charged. As the temperature increases, the corona voltage decreases, and the charge of the particles begins to increase significantly with time. At the same time, the charge of the particles increases with temperature. As the temperature increases, the average free path of electrons increases, the number of charged ions generated by collisions between electrons and neutral molecules increases and the frequency and probability of collision with charged ions further increase. As a result, the electric field charge of the particulate matter increases. The increase in temperature further enhances the intensity of the corona discharge, which will further increase the concentration of free electrons and charged ions in the electric field. The irregular thermal movement of the charged ions is affected by the temperature, and the diffuse charge of the particles further increases.

At the same time, the exhaust temperature affects the specific resistance value of carbon particles. When the temperature reaches above 100 °C, the charge transport of the particles mainly conducts through the interior of the particles. The volume specific resistance of the particles is negatively correlated with the temperature. The decrease of the specific resistance value could result in the more frequent move of the charge between the particles, which contributes to an increase in the charge of the particles. Therefore, a higher temperature will lead to an increase in particle charge and charge transfer, further increasing the signal output of the particle sensor.

### 4.2. Exhaust Gas Flow Rate

Similarly, the exhaust temperature in the sensor concentration test interval and the electrode voltage are set as 100 °C and 1000 V, respectively. The particle size and the concentration of particulate matter in the sensor are determined as 100 nm and 1.6 mg/m^3^, respectively. The exhaust gas velocity at the inlet 1 was selected as 5, 10, and 15 m/s. The regularity of the amount of charge of particulate matter flowing through the sensor with time is analyzed.

**Figure 6:**
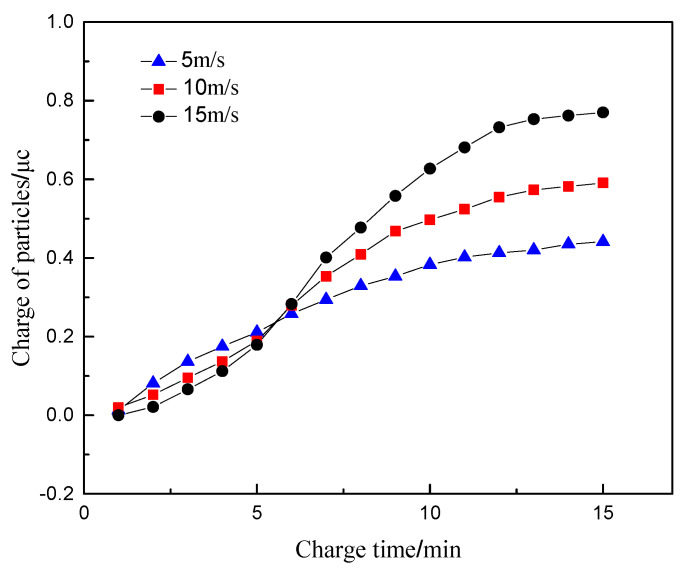
The relationship between the charge amount of particles and time at different flow rates.

Figure 6 shows the variation of the charge of particulate matter with time at different flow rates. It can be seen that within the first 6 min before charging, the higher exhaust flow rate brings about the smaller charge of particulate matter. This is because particles are mainly affected by the fluid drag force in the sensor, where their movement speed is mainly related to the fluid speed. The movement speed of the particulate matter also increases with the increase of the exhaust flow rate, which results in the reduction of the residence time of the particulate matter between the sensor plates. In other words, the charging time is reduced. Since the diffusion charge of particulate matter is mainly affected by the residence time of particulate matter in the electric field, the charge amount per unit of particulate matter shows a downward trend. When the charging time exceeds 6 min, the charge amount of the particulate matter increases with the increase of the exhaust flow rate. This is due to the increase of the amount of particulate matter flowing through the sensor concentration test interval in the same period. Therefore, with the increase of the charging time, the charge of the particulate matter generally increases with the increase of the exhaust flow rate, whose variation magnitude is not obvious.

### 4.3. Particle Size

The particle size of diesel exhaust particles is affected by the multiple effects of fuel combustion, particle oxidation and collision. The particle size varies with working conditions, which affects the charging characteristics of the particles. The exhaust temperature in the sensor concentration test zone, the electrode voltage, the concentration of particulate matter in the sensor, and the inlet method phase velocity are set as 100 °C, 1000 V, 1.6 mg/m^3^, and 10 m/s. The studied particle size parameters are selected as 20, 100, and 150 nm.

**Figure 7:**
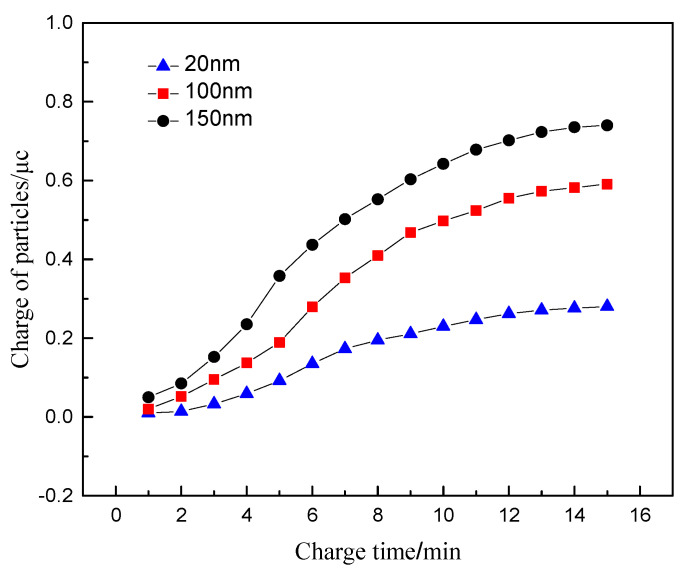
The relationship between the charge amount of particles with different particle sizes and time.

Figure 7 shows the variation of the charge of particles with different particle sizes with time. For particles flowing through the sensor concentration test interval, the charge of the particles increases with the increase of the particle size, showing a significant positive correlation. It can be seen from Equation (9) that when the particle size is less than 0.2 μm, the charging effect is mainly in the way of diffusion charging [16,24], which is not affected by the electric field charging effect. The particle size dp is proportional to the particle size. At the same time, the particles with the large size possesses the large surface area, which directly strengthens the ability to adsorb charged ions and cause a significant increase in the amount of charge.

### 4.4. Particle Concentration

**Figure 8:**
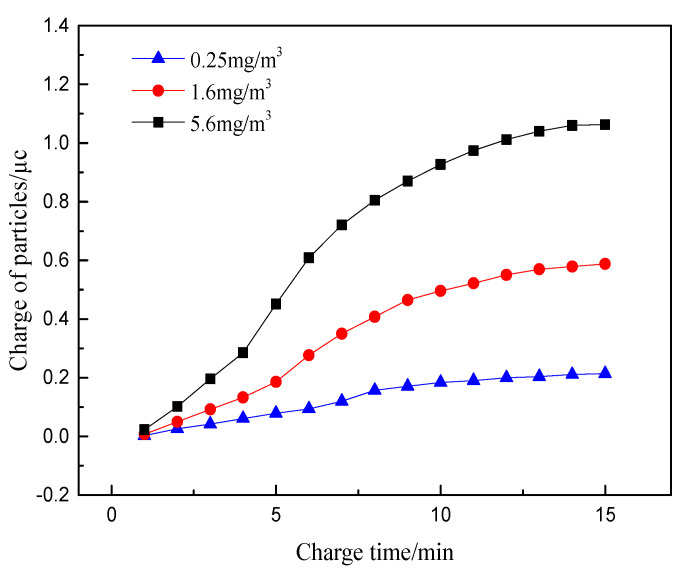
The relationship between the charge amount of particles and time at different concentration.

Changes in the operating conditions of diesel engines mainly cause changes in the concentration of particulate matter. The main purpose of the particulate matter sensor is to measure the exhaust gas concentration of the diesel engines in real time. By studying the charge amount of particles in different concentrations, the mechanism of concentration measurement of particle sensor is further studied, which is conducive to improve and optimize particulate matter sensor. The exhaust temperature in the sensor concentration test section, the electrode voltage, the exhaust flow rate, and the particle size are set as 100 °C, 1000 V, 10 m/s, and 100 nm, respectively. The studied particle concentrations are selected as 0.25 mg/m^3^, 1.6 mg/m^3^ and 5.6 mg/m^3^. The variation curve of the charge of particles with time under different concentrations is shown in Figure 8.

It can be seen that the charge of particulates increases obviously with the increase of particle concentration. This is attributed to the number of charged particles entering the electric field directly affecting the total charge of particles at high exhaust concentration. The space charge density caused by the concentration change has little effect on the electric field strength. The electric field charge and diffusion charge of the particles are not changed greatly with the change of particle concentration. However, the high concentration of charged particles could result in the more frequent collision motion after flowing through the electric field, an expression of further improving the charging effect and producing more obvious changes in the charge.

It can be seen that the charge of particulates increases obviously with the increase of particle concentration. This is attributed to the number of charged particles entering the electric field directly affecting the total charge of particles at high exhaust concentration. The space charge density caused by the concentration change has little effect on the electric field strength. The electric field charge and diffusion charge of the particles are not changed greatly with the change of particle concentration. However, the high concentration of charged particles could result in the more frequent collision motion after flowing through the electric field, in expression of further improving the charging effect and producing more obvious changes in the charge.

On the whole, with the continuous increase of exhaust gas temperature and particle concentration, the charge of particles increases obviously, while the increase of exhaust gas velocity and particle size has little influence on the charge increase of particles. Therefore, exhaust temperature and particle concentration are the main influencing factors of particle charge characteristics.

## 5. Sensor Charge Characteristic Test

In order to further study the influence of charge characteristics of particles on the output signal value of the sensor, a test bench was built by using the simulation experimental device to analyze the change of particle charge and the output law of the sensor signal under different working conditions. The charge change of the particulate matter and the sensor output signal under actual working conditions are analyzed through the engine bench test.

### 5.1. Text Programme

A test bench is established to verify the correctness of the simulation. The faraday tube in front of the sensor is used to measure the charge of the uncharged particulate matter to ensure that the total charge of the measured particulate matter is electrically neutral. The charge of the particulate matter flowing through the sensor is measured to obtain the change of the charge of the particulate matter flowing through the sensor under the conditions of different gas temperature, different gas flow rate and different particle size. Since the output signal value of the sensor is mainly positively correlated with the concentration of particulate matter, the exhaust state and the particle size of the particulate matter could affect the signal output of the sensor signal at the same concentration, resulting in a deviation in the output signal value. On this basis, the sensor signal output test is carried out synchronously. Two identical particulate sensors are selected. The particulate signal output at any operating point is repeatedly collected four times, and the four measurement results are averaged to obtain the sensor signal at the changing relationship under different test conditions. This provides data support for the next calibration study. The test is divided into four parts as follows.

The exhaust temperature, the particle size, and the particle concentration are set as 100 °C, 100 nm, and 1.6 mg/m^3^. The inlet exhaust flow rate is determined as 5 m/s, 10 m/s, 15 m/s, and 20 m/s through the flow adjustment system. The digital charge meter is used to measure the charge of the particles in the faraday tube behind the sensor after turning on the particle generator for 5 min. Meanwhile, the output signal value of the sensor is also measured.

The exhaust gas flow rate, the particle size and the particle concentration are set as 10 m/s, 100 nm, 1.6 mg/m^3^, respectively. The exhaust temperature is determined as 50 °C, 100 °C, 125 °C and 150 °C through the pipe heater. The digital charge meter is used to measure the charge of the particles in the faraday tube behind the sensor after turning on the particle generator for 5 min. The output signal value of the sensor is also measured.

The exhaust flow rate, the exhaust temperature and the particle concentration are set as 10 m/s, 100 °C and 1.6 mg/m^3^, respectively. The size of the particles is determined as 20 nm, 50 nm, 100 nm, and 150 nm. The digital charge meter is used to measure the charge of the particles in the faraday tube behind the sensor after turning on the particle generator for 5 min. The output signal value of the sensor was also measured.

The engine bench test is carried out. The torques of 10 N·m, 50 N·m, 75 N·m and 100 N·m at the speed of 1000 r/min are selected as test conditions. The particle charge and the output signal of the sensor under the test conditions are measured. The digital charge meter is used to measure the charge of the particles in the Faraday tube behind the sensor after turning on the particle generator for 5 min. The output signal value of the sensor is also measured.

### 5.2. Test System and Equipment

#### 5.2.1. Simulation Test Bench

**Figure 9:**
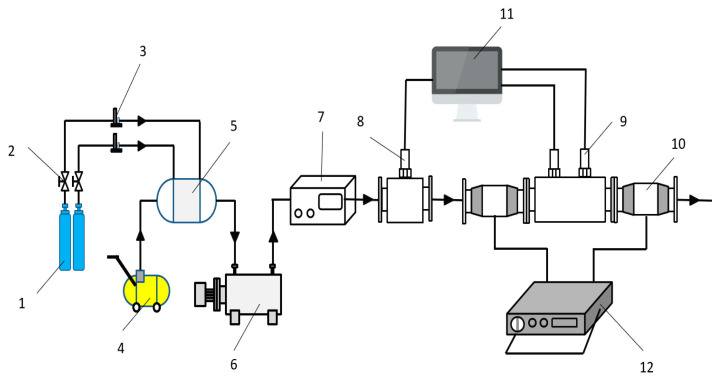
Simulation test bench for charge performance of particles. (1) Nitrogen cylinder (2) Throttle valve (3) Flow meter (4) Air compressor (5) Pressure stabilizing cylinder (6) Pipeline heater (7) Particle generator (8) Pressure sensor (9) Leakage current particulate matter sensor (10) Faraday cylinder (11) Computer (12) Digital charge meter.

**Figure 10:**
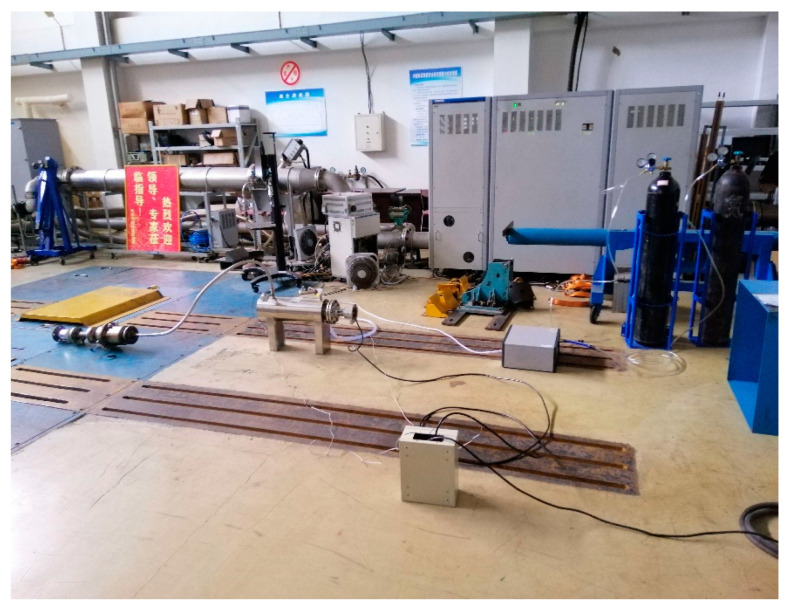
Experimental layout of Simulation test bench.

A simulation test beach, which can simultaneously measure the charge of particles and the signal of particle sensor, is built according to the requirements of the test. As shown in Figure 9 and Figure 10, the bench is mainly composed of exhaust simulation system, charge measurement system and sensor signal test system.

In the exhaust simulation system, nitrogen is used as the particle carrier. The gas in the nitrogen cylinder flows into the pipeline heater through the pressure stabilizing tube, and is heated to the temperature required by the test, then flows into the particle generator. The particle generator generates the particle size required by the test which can be used to simulate the exhaust with controllable temperature and particle size after mixing with nitrogen. Finally, the gas is passed into the test system. The back pressure of the gas can be obtained by the pressure sensor. The velocity of the exhaust can be obtained by the ideal gas state equation. The test system is mainly composed of charge measurement system and sensor signal test system. The charge measurement system mainly relies on the Faraday tube and digital charge meter to measure the change of charge amount of particles. The sensor signal test system uses two sensors to measure the change of output signal value of particles under different conditions.

#### 5.2.2. Particulate Matter Generator

DNP3000 particle generator produced by German PALA company was used to produce the particles needed for the test. The generator is able to adjust the generated carbon soot particles rapidly, and the main parameters are shown in Table 1. The particle generator generates condensed aerosol through high pressure flashover between two graphite electrodes, which is similar to diesel exhaust particles in shape, size, density, surface morphology and refractive index. With flashover, a small amount of graphite material is removed from the high pressure electrode at high temperature and condenses to form the smallest particles. The particle size of primary particles is 3–5 nm, and due to the high quantity concentration, these small particles will agglomerate into clumps. The particle size after agglomeration is 10–150 nm, and the formation of agglomeration can be adjusted in a certain way. After mixing air, the gas particles with a certain concentration are formed to simulate diesel exhaust. Due to a constant flashover voltage, the energy converted in each spark remains constant. Therefore, the particle size distribution and agglomeration amount of particles are also different with the frequency of electric spark. The stability of aerosol generation can be guaranteed by strictly controlling the distance between electrodes in the flashover process, and the quantity and mass flow of particulate can be rapidly regulated by adjusting the spark frequency. The generator is calibrated by particle size spectrometer for engine exhaust emissions (EEPS).

#### 5.2.3. Charge Measuring System

**Figure 11:**
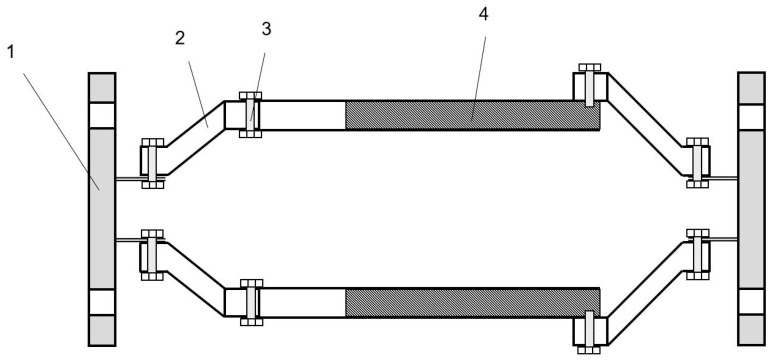
Structure of Faraday cylinder. (1) Flange (2) PVDF reducer (3) Fastener (4) Insulating rubber.

The charging measurement system consists of Faraday tube and EST111A digital charge meter. The Faraday tube needs to be modified in order to facilitate the charge measurement in the test process. The modified Faraday cylinder shall meet the following conditions: (1) it has certain high-temperature and high-pressure resistance performance; (2) good air tightness; (3) high accuracy of charge measurement. The modified Faraday cylinder is shown in Figure 11.

The prototype of the Faraday tube is a cylindrical vessel consisting of two coaxial cylindrical tubes and an insulating rubber between the layers. The working principle is as follows: the particles will be collected by the inner cylinder when the charged particles enter the Faraday cylinder. The outer cylinder is completely isolated by the insulator and connected to the digital charge meter through the interface, and it is grounded to keep the electrostatic field shielded. When the particles enter the Faraday cylinder, according to the principle of electrostatic induction, the outer surface of the inner cylinder and the inner surface of the outer cylinder will have the same amount of heterogeneous charges. The charge amount of the particles can be obtained by measuring the induced charges generated by the particles entering the sensor. In order to load the Faraday cylinder directly into the test bench, the bottom of the original Faraday cylinder was cut off, and the large and small head made of polyfluoroethylene material was connected with the flange plate. At the same time, all parts were connected with fasteners, and the sealing place was coated with insulation glue, so as to ensure that it had good performance of high temperature and high pressure resistance and air tightness. A glass fiber filter film filter paper is installed at the internal outlet end of the Faraday tube behind the sensor to capture the charged particles entering the tube. Two Faraday tubes were connected to the particle sensor respectively, and the variation trend of the cumulative charge of particles after being charged by the high-voltage electrode of the sensor could be obtained.

#### 5.2.4. Pipe Electric Heater

Pipe electric heater is a new type of heating equipment developed by Maiheng Electric Equipment Company in recent years. The OCr27A17MO2 high temperature resistance alloy wire and crystalline magnesium oxide powder are formed through compression process, so that the service life of the electric heating element can be guaranteed. The control part is composed of adjustable temperature measuring and constant temperature system with high-precision digital display temperature controller, which ensures the normal operation of electric heater. Scope of application: ammonia heater, air heater, water pipe heater, other kinds of gas, liquid pipe heating equipment.

**Figure 12:**
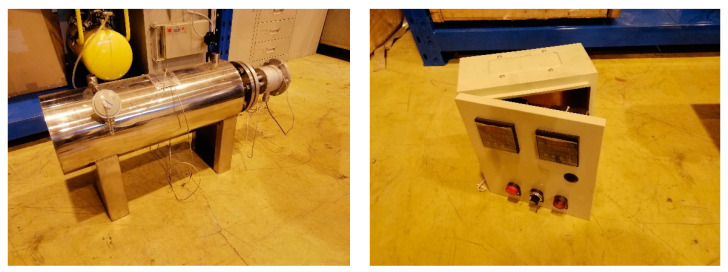
Pipe electric heater and control box.

Pipe electric heater adopts digital display temperature control instrument, solid state relay and temperature measuring element composition measurement, adjustment, control circuit, temperature measurement in the process of electric heating element will pipe outlet temperature of the electric heater electrical signals sent to the digital display temperature control instrument of amplification, after comparing measured temperature value, at the same time the output signal to the solid state relay input, so as to control the heater, the controller has good control precision and regulating characteristics. The interlock device can be used to start and close the electric heater of pipeline remotely.The pipe electric heater and control box used in the test are shown in Figure 12.

### 5.3. Analysis of Test Results

#### 5.3.1. Charge Characteristics of Particles and Sensor Signal Output at Different Flow Rates

**Figure 13:**
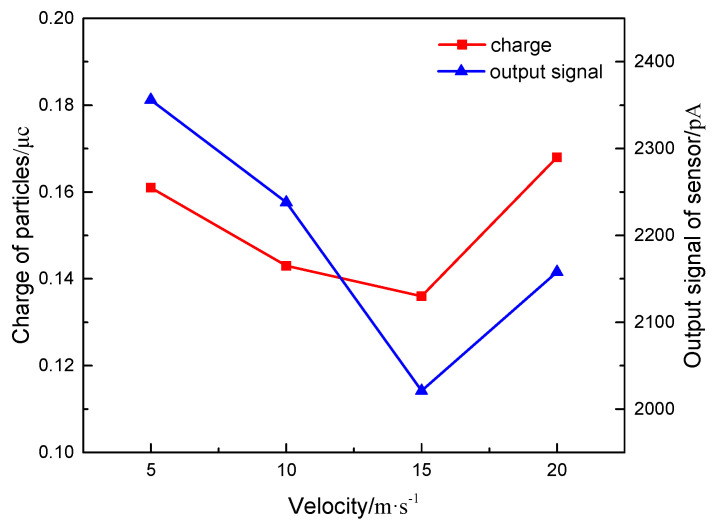
The relationship of charge of particles and output signal of sensor changing with different exhaust velocity.

Figure 13 shows the relationship between the particle charge and the output signal value of the sensor with the exhaust flow rate. It can be seen from the figure that when the exhaust flow rate increases, the charge of particles decreases first and then increases. It can be known that the increase of flow rate reduces the charging time of particles, thus reducing the charge of particles. Meanwhile, it increases the number of charged particles per unit time. When the exhaust flows at a low rate, few charged particles enter the concentration test section. The charge of single particle is the main factor affecting the total charge. Therefore, the amount of charge decreases as the exhaust flow rate increases. With the increase of exhaust flow rate to a certain value, the number of charged particles increases greatly in unit time. At this time, the number of charged particles takes main part in affecting the charge and thus the charge of particles begins to increase. Only some particles enter the sensor during the test, contributing to the implicit effect of flow rate on the charge of particles.

The output signal value of the sensor is similar to the change of the charge of the particulate matter, which shows the law of decreasing first and then increasing. This is mainly because the charge transmission of the particles in the sensor directly affects the signal output of the sensor. When exhaust flows at a low rate, the particles possess the large charge, and they remain between the plates for a long time. This directly leads to sufficient charge transfer. With the increase of flow rate, the particle charge decreases, and the output signal value of the sensor begins to decrease. When the flow rate increases to a certain stage, the charge of the particulate matter and the output signal value of the sensor exhibit the opposite trend with the above-mentioned law. However, the short retention time of particles in the sensor could result in that some particles do not contact the grounding electrode. Thus, those particles flow out of the concentration test range, which is not conducive to the generation of the output signal of the sensor.

#### 5.3.2. Charge Characteristics of Particles and Sensor Signal Output at Different Temperatures

**Figure 14:**
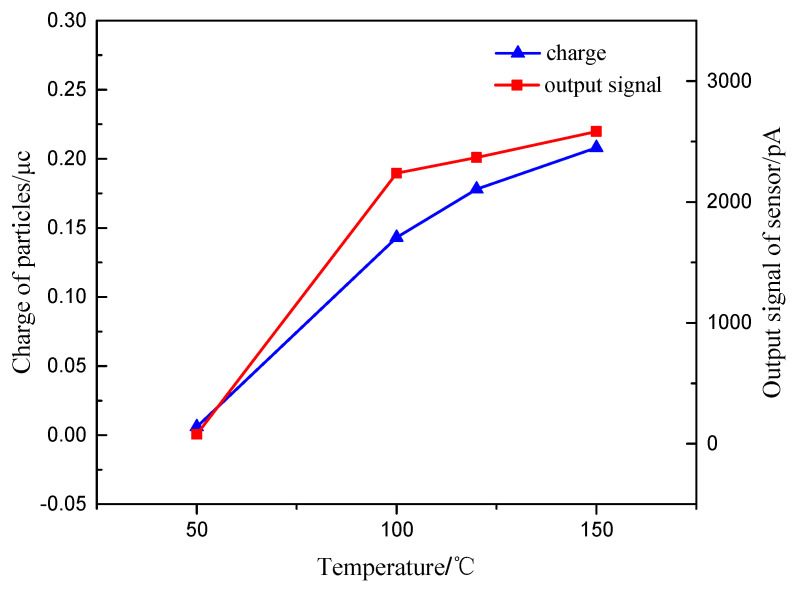
The relationship of charge of particles and output signal of sensor changing with different exhaust temperature.

Figure 14 shows the relationship between the charge of particles and the output signal value of the sensor with the exhaust temperature. It can be seen that when the temperature is too low to reach the halo temperature of the high-voltage electrode, the charge of particles is very small or even close to zero. With the increase of exhaust temperature, the charge of particles exhibits an obvious growth trend. It can be explained that the increase of temperature can increase the electron stroke and discharge intensity, which increase the concentration of charged ions in the concentration test range, intensify the collision and adsorption of particles and charged ions, reduce the specific resistance of particles, and increase the electric field charge and diffusion charge of particles.

The output signal value of the sensor increases with the increase of the exhaust temperature. This is because the increase of temperature not only increases the charge amount of particles, but also strengthens the movement of particles between the plates, which is conducive to the diffusion charge of particles. Meanwhile, the retention charge effect of particles in the concentration test range could be neglected. This can assist the charge transfer of particles between electrode plates and the generation of sensor signal. At the same time, the increase of exhaust temperature leads to the increase of relative permittivity of exhaust gas. The signal value of sensor leakage current is positively related to the relative permittivity of exhaust gas. The increase of exhaust relative permittivity increases the leakage current value of sensor. Thus, the output signal value of the sensor increases with the exhaust gas temperature.

#### 5.3.3. Charge Characteristics of Particles and Sensor Signal Output with Different Particle Sizes

**Figure 15:**
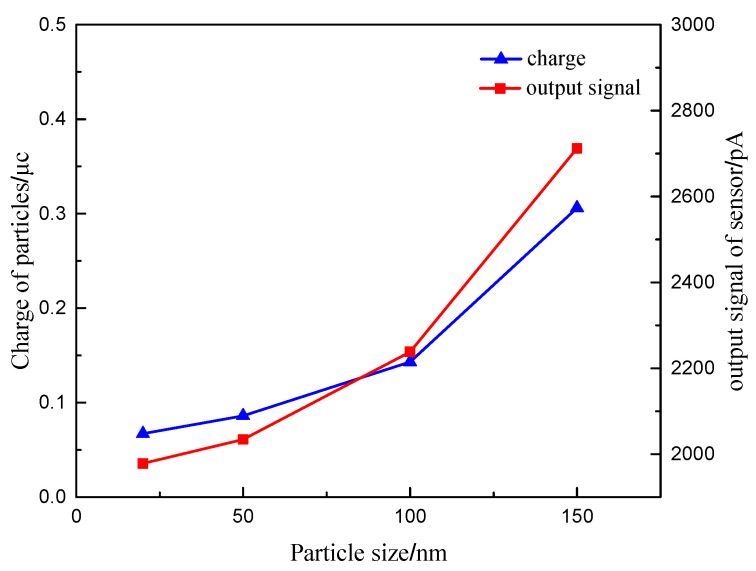
The law of charge of particles and output signal of sensor changing with different particle sizes.

Figure 15 shows the relationship between the particle charge and the output signal value of the sensor with the particle size. It can be seen that the charge of the particles with the increase of particle size, due to the obvious positive correlation between the charge of particles and the particle size. At the same time, the more obvious movement trend of particles with large size results in more frequent particle collisions, and leads to the increase of charge in the identical time.

With the increase of particle size, the output signal value of the sensor increases, and the growth trend is more and more obvious. It can be seen that the output signal value of the sensor is basically consistent with the change rule of the charge. This is because the particles with the large particle size in the concentration test section can be more affected by the electric field force. Meanwhile, due to the increase of the particle size, the exhaust drag force on the particles increases, which lead to the more obvious trend of the migration to the grounding electrode. The migration of the particles assists in the improvement of the charge transmission between the electrode plates and increases the sensitivity of the sensor signal output.

#### 5.3.4. Charge Characteristics of Particles and Sensor Signal Output with Different Working Conditions

**Figure 16:**
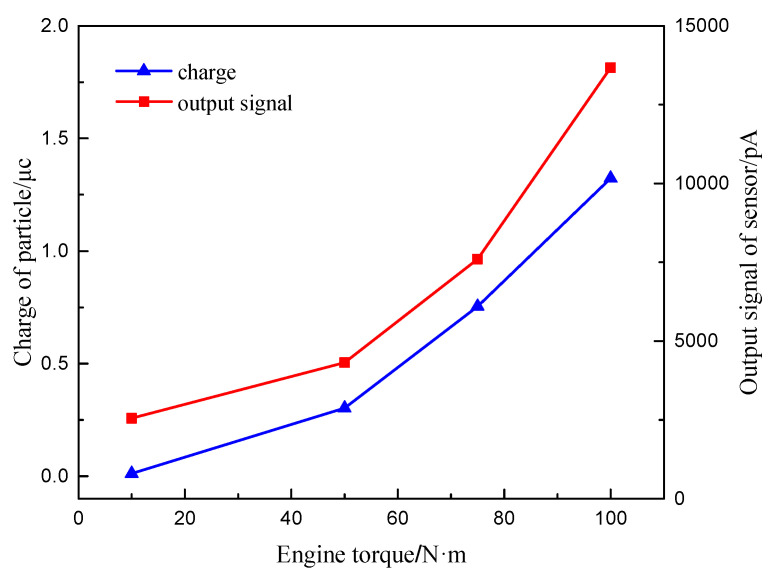
The law of charge of particles and output signal of sensor changing with different engine torque.

Figure 16 shows the relationship between the particle charge and sensor output signal value with different engine torques of 1000 r/min. It can be seen that the charge of the particulate matter increases with the increase of engine torque, because the engine exhaust temperature increases with the increase of the engine torque. The exhaust temperatures under the four operating conditions are 75 °C, 135 °C and 220 °C, respectively. The increase of load leads to the increase of intake flow and exhaust flow rate. Meanwhile, the particle concentration, the median particle size and the surface area of the accumulated and nucleated particles increase with increase of load [25]. It can be known from the simulation test data that the increase of exhaust temperature, exhaust flow rate and particle size contribute to the increase of the particle charge. Therefore, the charge of the particle increases with engine torque.

The output signal value of the particulate matter sensor also increases with the increase of engine torque, whose growth trend is obvious. This is because as the engine load increases, the section of high concentration mixed fuel increases, resulting in insufficient combustion and increasing exhaust smoke. This directly increases the output signal value of the sensor. Meanwhile, the increase in the charge of the particulate matter also further increases the output signal value of the sensor.

## 6. Conclusions

According to the exhaust state of the engine, the boundary conditions were determined to conduct multi-physical field and particle charge simulation, and the simulation results were verified by the simulation test bench, and the following conclusions were obtained:

(1) After the exhaust gas inside the exhaust pipe flows through the sensor, an obvious velocity stratification occurs, and the flow velocity on the leeward side of the sensor declines significantly. A slight variation in the exhaust flow velocity occurs at the lower end of the sensor leeward side. The gas flow velocity inside the sensor drops rapidly from the inlet which leading to the gradient distribution of the flow velocity and remains stable after entering the protection zone outside the sensor. The electric field intensity in the concentration test section decreases gradually from the high voltage electrode to the grounding electrode.
(2) The increase of gas temperature enhances the intensity of corona discharge, intensifies the movement of particles, and makes the collision between particles and charged ions more frequent. Meanwhile, the specific resistance of particles is reduced leading to improve the ability of particles to collect charge, and increase the charge of particles. Because the relative dielectric constant of exhaust gas, which has a positive correlation with the signal output of the sensor, grows with the increase of temperature, the output signal value of the sensor increases as well.
(3) The charge of particles is reduced with the increase of the gas velocity because of the reduction of charge time of particles. However, the number of particles flowing through the sensor increases and lead to increase of the total charge of particles. Therefore, the charge of particles grows with the increase of flow velocity after a certain charge time. When the charging time exceeds 5 min, the output signal value of the sensor decreases first and then increases with the exhaust gas flow rate.
(4) The charge of particles is directly proportional to the particle size. Particles with larger particle sizes tend to have larger surface areas which result in the stronger ability to absorb the charge. The sensor output signal value grows as the particle size increases as well. As the concentration of particles increases, the charge of the particles shows a clear upward trend, and the increasing trend of the charge of the particles becomes more obvious as the charging time increases, the sensor output signal value also grows with the increase of the particulate matter concentration.

A further study will be performed for commercial application in the future. It is necessary to calibrate the output signal value of the sensor under different working conditions to ensure the accuracy of the measured concentration of particulate matter.

## Figures and Tables

**Table 1:** Main technical parameters of dnp3000 particle generator.

Technical Indicators	Numerical Value
Particulate materials	Carbon; Others can be provided upon request
The carrier gas	nitrogen
Initial pressure	4–6 bar
Volume flow	0–40 L/min
The number of traffic	>10^7^ P/cm^3^
Mass flow rate	0.06–25 mg/h
Particle size	Junior 3–5 nm
	Reunion 10–150 nm
